# Une tumeur historique de la fesse: à propos d’un cas

**DOI:** 10.11604/pamj.2017.27.39.10914

**Published:** 2017-05-15

**Authors:** Hasnaa Zaouri, Baderdine Hassam

**Affiliations:** 1 Service de Dermatologie, CHU Ibn Sina, Rabat-Instituts, Rue Famfdal Cherkaoui, BP 6527, 10000 Rabat, Maroc

**Keywords:** Lymphome anaplasique, lymphome T CD30, fesse, Anaplastic lymphoma, T-cell lymphoma CD30+, buttock

## Image en médecine

Le lymphome anaplasique à grandes cellules (LAGC) est un type rare de lymphome T. Il représente environ 1 à 2 % de tous les cas de lymphome non hodgkinien (LNH) chez l'adulte. Nous rapportons le cas d'une tumeur historique de la fesse Nous rapportons le cas d'un patient âgé de 48 ans, sans antécédents pathologiques notables, qui s'est présenté pour un placard tumoral de la fesse droite, évoluant depuis 12 ans. L'examen dermatologique retrouvait un placard tumoral de 15 cm de diamètre fait de nodules tumoraux érythémato-violacés, dont certains sont ulcérés, siège au niveau de la fesse droite. Avec un nodule controlatéral .le reste de l'examen clinique était sans particularités. L'étude immuno-histologique était en faveur d'un lymphome T anaplasique CD30 +.Le bilan d'extension a objectivé des adénopathies inguinales et iliaque externe d'allure lymphomateuse. Une exérèse chirugicale après une chimiothérapie CHOP était indiquée. Notre observation souligne l'importance de la sensibilisation de la population pour un diagnostic et prise en charge précoce.

**Figure 1:**
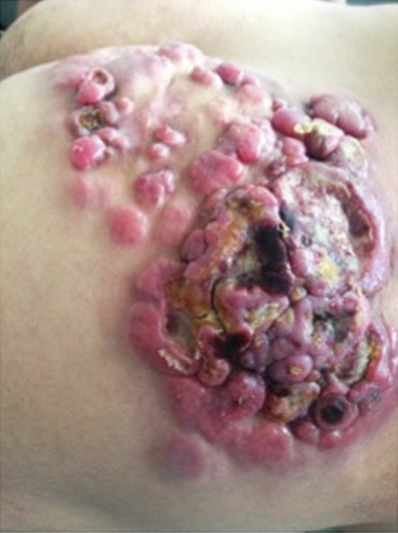
Placard tumoral de la fesse droite

